# Hermetic storage of wheat and maize flour protects against red flour beetle (*Tribolium castaneum* Herbst)

**DOI:** 10.1371/journal.pone.0185386

**Published:** 2017-09-26

**Authors:** Yan Yan, Scott B. Williams, Larry L. Murdock, Dieudonne Baributsa

**Affiliations:** 1 Department of Biological Sciences, Vanderbilt University, Nashville, Tennessee, United States of America; 2 Spensa Technologies, INC., West Lafayette, Indiana, United States of America; 3 Department of Entomology, Purdue University, West Lafayette, Indiana, United States of America; US Department of Agriculture, UNITED STATES

## Abstract

Hermetic storage is used to protect grain against insect pests, but its utility is not limited to whole grains. We evaluated hermetically-sealed, polyethylene terephthalate (PET) bottles for preserving wheat and maize flour against red flour beetle (RFB, *Tribolium castaneum*, Herbst) population growth. Flours infested with RFB and kept in sealed PET bottles experienced much less weight loss over a three-month storage period than infested flour kept in unsealed bottles. RFB populations in wheat flour kept in sealed bottles did not increase, while populations in unsealed bottles grew about 50-fold during the same three-month period. Flour in sealed bottles had lower levels of oxygen and moisture than flour stored in unsealed bottles. Similar trends were observed for oxygen and moisture levels in maize flour held in hermetically sealed bottles. Hermetically-sealed bottles were effective in preventing RFB population growth and preserving maize and wheat flour. Farmers, consumers and food processors can safely store grain flour in hermetic sealed containers.

## Introduction

Red flour beetle (RFB), *Tribolium castaneum* (Herbst) (Coleoptera: Tenebrionidae), is a major economic pest of flour and other processed food products [1–3]. In developing countries, poor storage and handling practices by smallholder farmers make it easier for insects to infest stored grain [4]. Maize and wheat are the most important cereal grains grown worldwide and their flour products make up a significant portion of the diet for people in Africa [5,6]. Insect infestations cause a reduction in grain weight, lower nutritional and economic value, and reduced seed germination. Damaged grains may no longer be suitable for human consumption [7].

Fumigants have been routinely used to control insect populations in stored products. The major fumigants used worldwide have been phosphine and methyl bromide. However, these chemical control methods are becoming less effective due to the development of resistance and are subject to environmental concerns. New management alternatives are needed [8–13].

Modified atmospheres (MA) are effective and non-chemical alternatives to fumigants for controlling insect pests in stored grains [14,15]. Hermetic storage is a type of MA control in which the MA is generated by respiration of insects and grain in an airtight container. Biological activity depletes oxygen (hypoxia) and elevates the level of CO_2_ (hypercarbia). With inadequate oxygen available, insect development and reproduction is limited, mortality rates increase, and damage to the stored grain is prevented [11,16–18]. MA technology has been shown to maintain grain quality against insect pests and arrest fungal growth [4,19,20].

Given its effectiveness in protecting grain against insect pests, hermetic storage has not been tested on wheat and maize flour in a controlled setting. Specifically, its effectiveness in preventing destructive RFB populations from developing in flours has not been demonstrated, although there is evidence from short-term studies that suggests hermetic conditions would be effective. In the present study, we assessed the effectiveness of hermetic storage using polyethylene terephthalate bottles (PET) in protecting maize and wheat flour against the RFB.

## Materials and methods

Red flour beetles (*Tribolium castaneum* Herbst) were obtained from colonies maintained on wheat flour by the Purdue Improved Crop Storage (PICS) Laboratory at the Department of Entomology, Purdue University. The colonies were kept in a Conviron^™^ Environmental Chamber (C710) (Winnipeg, Canada) at 25°C and 40% R.H. The wheat flour used in this study was purchased from Monarch (Rosemont, Illinois, USA); the maize flour from Bob’s Red Mill (Milwaukie, Oregon, USA). Both flours are commercial grade products of fine quality with initial moisture contents at 4.79% for wheat and 5.59% for maize.

Transparent water bottles- 26 cm tall and one liter capacity each- made of polyethylene terephthalate (PET, Glaceau Smartwater) were purchased from Wal-Mart Corp. Three fluorescent yellow Oxydots (Oxydots, OxySense^®^, Las Vegas, Nevada, USA) were attached to the inside wall of each bottle. The upper Oxydot was placed near the top, on the tapered portion of the bottle approximately 2 cm from the neck. The remaining two oxydots were placed at 6 cm (middle) and 24 cm (bottom) from the neck of the bottle.

Treatments were divided into groups differing as to whether the bottles were hermetically sealed and infested with RFB. This resulted in one treatment group (sealed-infested) and three control groups (unsealed-infested, sealed-uninfested and unsealed-uninfested) for each flour type. There were three replicates of each treatment. Each bottle was loaded with 400g of either maize flour or wheat flour. The flour covered all but the uppermost Oxydot. Infested bottles were each infested with 125 adult RFB per kg of flour. Sealed bottles had their caps tightly screwed into place and then the caps were wrapped with three layers of Parafilm around the outer edge to ensure a tight seal. The mouths of unsealed bottles were covered with cloth mesh secured with rubber bands. All treatments were held for three months (91 days) at 25°C and 40% R.H. in an environmental chamber.

Oxygen concentrations in each bottle were recorded daily for the first two weeks and weekly thereafter. At the end of three months, the following assessments were carried out: (1) the number of adult RFB present; (2) percentage loss of flour; (3) the moisture content of the flour.

At the end of three months, we sealed all bottles with three layers of Parafilm to ensure there was no moisture loss and froze the bottles at -20°C for two days to kill any insects present. We then separated the adult insects and flour portions from the infested bottles with two #20 sieves (Opening size: 0.84 mm) and one collecting pan stacked together, all purchased from Cole Parmer (Vernon Hills, IL). Two sieves were used to ensure that larvae were excluded from the sieved flour as much as possible. Samples were collected and weighed separately. We also counted the number of adult RFB. Immature stages were not quantified due to their range in sizes and the difficulty of separating them completely from the flour.

To determine moisture content of the flour, we used a modified methodology based on AACC method 44–15.02 [21]. We filled plastic petri dishes (100 x 15 mm, VWR, Radnor, PA, USA) with the sifted flour from each bottle until it was flush with the upper rim of the dish. We weighed the wet mass of each dish with flour, placed them in a drying oven at 60°C for four days and then weighted the dry mass of each dish. The change in the mass was used to calculate the moisture percentage of the flour. Three samples were taken from each bottle.

Statistics were performed using GraphPad Prism 7.01 (GraphPad Software, Inc.). Two-factor ANOVA was used for all statistical comparisons except where noted otherwise.

## Results

### Internal oxygen levels

**Fig 1:**
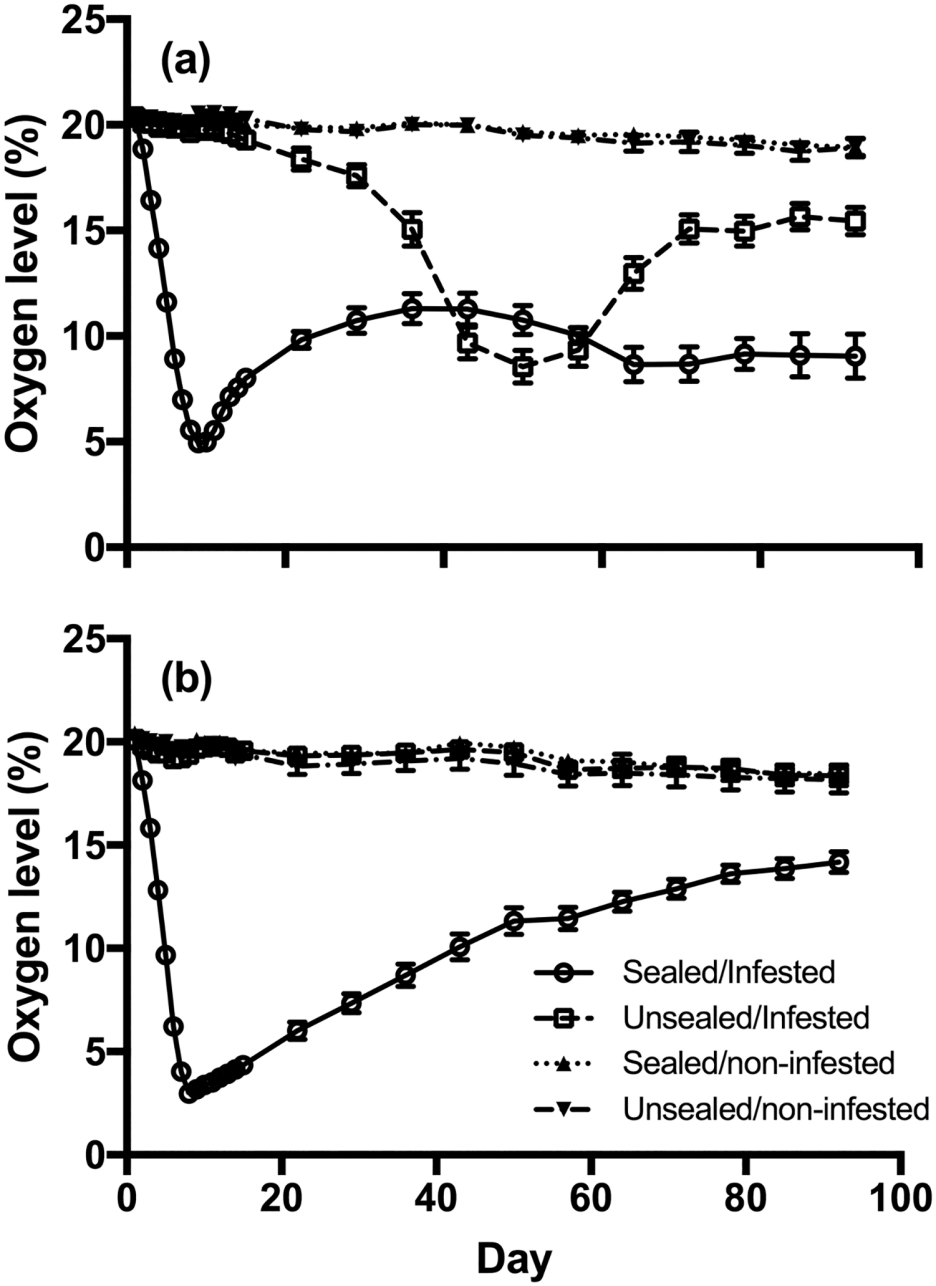
Dynamics of oxygen levels within PET-bottles containing wheat flour (a) and maize-flour (b) over time. Oxygen readings from sealed infested bottles showed different trends than those observed in unsealed bottles (wheat flour: F_(3, 32)_ = 454.5, P < 0.0001; maize flour: F_(3, 32)_ = 374.3, P < 0.0001). Each data point is the average of the oxygen levels recorded from three Oxydots (upper, middle and bottom) in all three replicates for that treatment. Error bars represent the standard error of the mean.

Hermetic sealing had a significant effect on internal oxygen levels in the bottles (wheat flour: F_(3, 32)_ = 454.5, P < 0.0001; maize flour: F_(3, 32)_ = 374.3, P < 0.0001) (Fig 1). When the flour was infested with RFB, the oxygen levels in the sealed bottles were generally lower on average than in unsealed bottles across the three-month study period. Oxygen levels fell rapidly in sealed bottles, dropping to as low as 5% (v/v) (wheat) and 3% (v/v) (maize) oxygen within the first week of the study. Over the next three months, oxygen levels in infested sealed bottles with both types of flour gradually increased until they reached between 10–15% (v/v).

Oxygen levels in unsealed, infested bottles were different for the two flour types (Paired t-test: t = 2.993, df = 25, P = 0.0061) (Fig 1). In the unsealed bottles containing wheat flour with insects present, the oxygen levels slowly decreased to 17% (v/v) during the first month, and then dropped more rapidly to between 8–10% (v/v) by the 50 day mark (Fig 1a). Levels slowly returned to between 15 and 17% (v/v) during the third month of the study. Oxygen levels in sealed bottles with no insects present remained stable around 20% (v/v) for all three months in bottles containing wheat or maize flour.

Oxygen levels were different depending upon the position of the Oxydot in the bottle. Oxygen levels recorded from the upper dots were higher than those recorded from the middle (Mean difference = 0.94% (v/v), paired t-test: t = 14.66, df = 207, P < 0.0001) and bottom dots (Mean difference = 1.07% (v/v), paired t-test: t = 14.66, df = 207, P < 0.0001) that were surrounded by flour. Oxygen levels between the middle and bottom dots were significantly different (Mean difference = 0.12% (v/v), paired t-test: t = 4.74, df = 207, P < 0.0001).

### Population size

**Fig 2:**
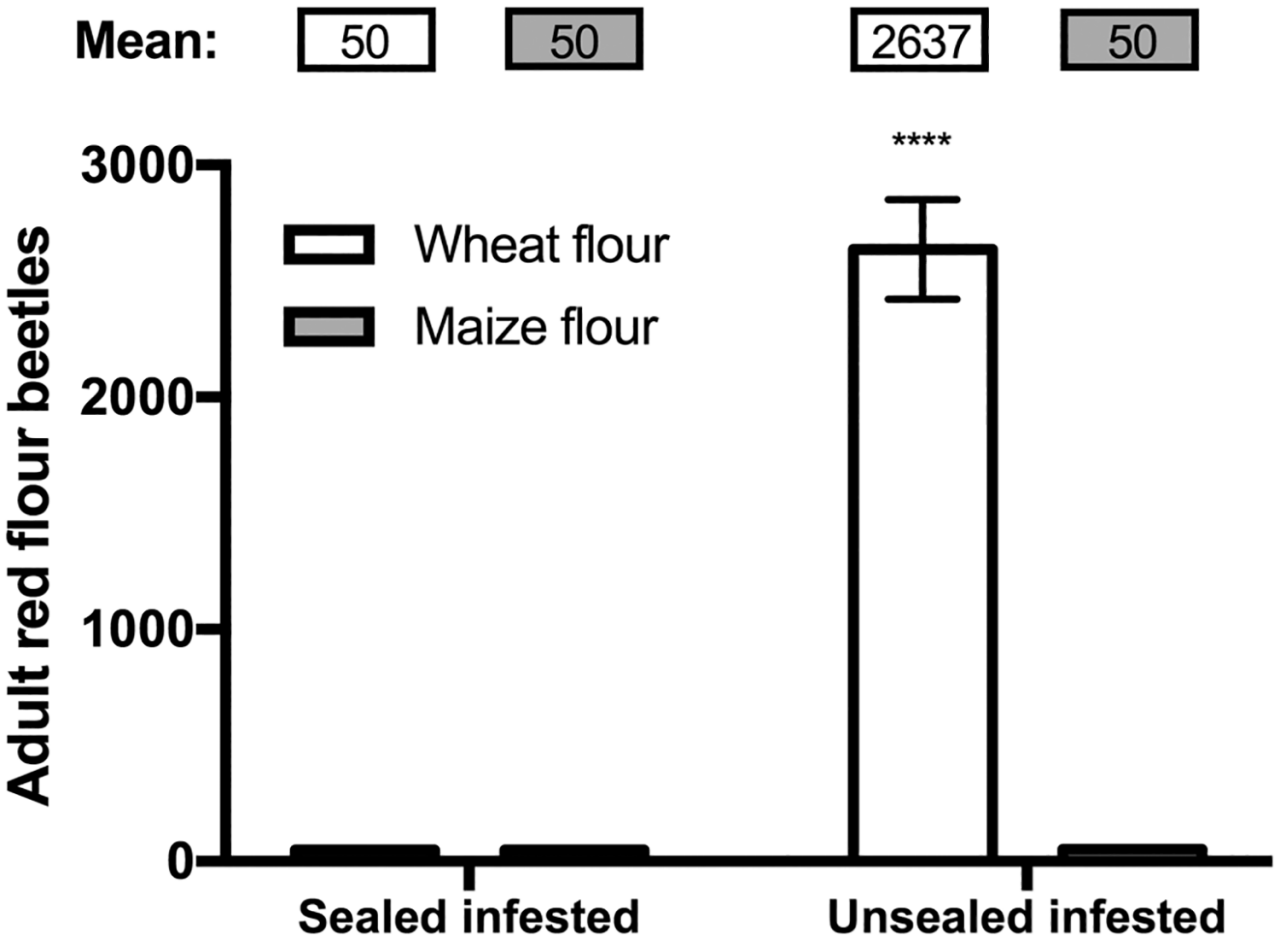
Adult red flour beetle (RFB) population sizes after three months under different storage conditions. The adult populations found in wheat flour in the unsealed infested bottles increased some 5-fold after three months, while the adult populations in other infested groups remained the same as was present initially (Sealed condition: F_(1, 8)_ = 144, P < 0.001; Flour type: F_(1, 8)_ = 143.7, P < 0.0001). Column heights show the mean and the error bars represent the standard error of the mean. “****” represents P < 0.0001.

Adult RFB population sizes after three months were significantly affected by the treatment condition (Sealed condition: F_(1, 8)_ = 144, P < 0.001; Flour type: F_(1, 8)_ = 143.7, P < 0.0001) (Fig 2). In sealed bottles, the size of the adult population was unchanged at the end of the experiment compared to the initial infestation level (50 ± 0 RFB). We observed that all beetles inside sealed bottles had stopped moving two week after we started the experiment. By contrast, the RFB populations in wheat flours in the unsealed bottles increased dramatically, reaching 2636 ± 215 within three months. Interestingly, the number of adult beetles in unsealed bottles containing maize flour did not increase over the storage period. Even so, we observed large numbers of larvae in the unsealed bottles for both maize and wheat flour. We observed that larvae feeding on maize flour exhibited a red tint.

### Flour weight loss

**Fig 3:**
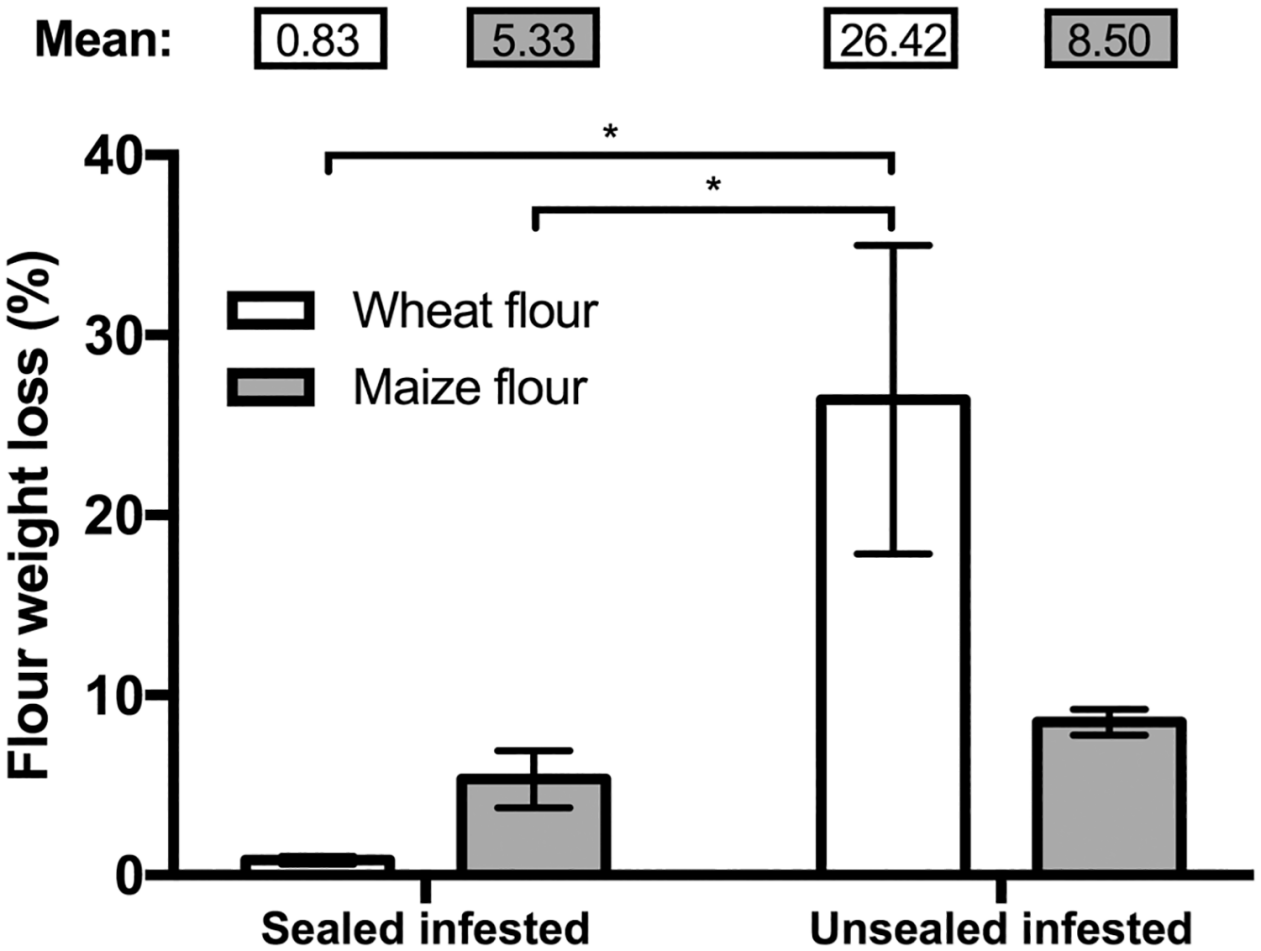
Flour weight loss caused by red flour beetle (RFB). Sealed infested bottles saw little weight loss over the study period, while loss of wheat flour mass in the unsealed infested group was significantly higher than in the other infested groups (Sealed condition: F_(1, 8)_ = 10.79, P = 0.0111; Flour type: F_(1, 8)_ = 2.35, P = 0.1638). Column heights represent the mean and the error bars represent the standard error of the mean. “*” represents P < 0.05.

There was no change in mass of both flour types for the sealed infested group. At the same time, the unsealed, infested bottles with wheat flour experienced large weight losses (26.42 ± 8.57% (v/v)) relative to the other treatment groups (Sealed condition: F_(1, 8)_ = 10.79, P = 0.0111; Flour type: F_(1, 8)_ = 2.35, P = 0.1638) (Fig 3). Flour weight did not significantly change in any of the non-infested bottles after three months of storage.

### Flour moisture content

**Fig 4:**
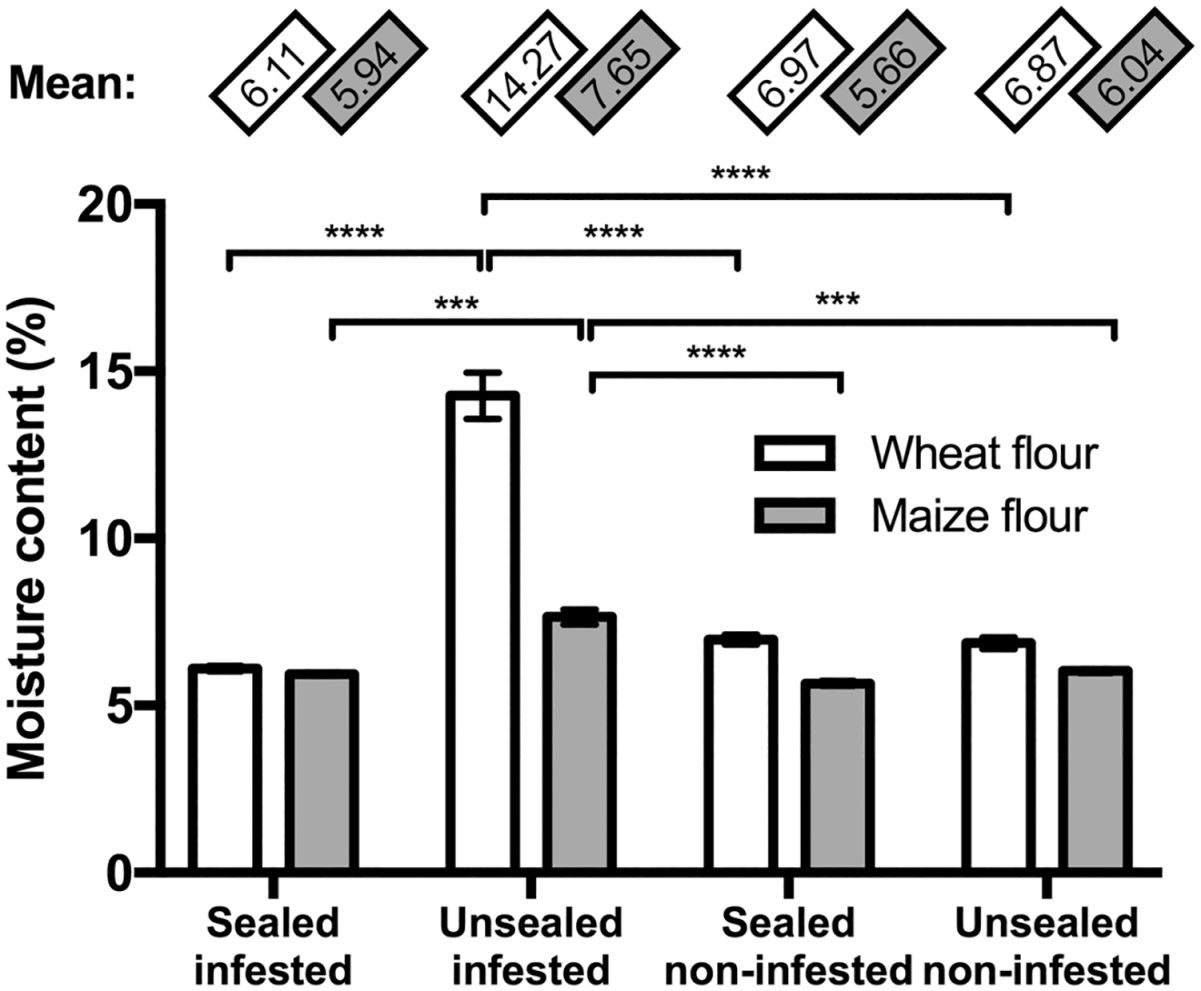
The treatment had a significant effect on the moisture content of the flour. The unsealed infested treatment showed a higher moisture reading than the ones within each flour type (Sealed and infestation condition: F_(3, 64)_ = 147.3, P < 0.0001; Flour type: F_(1, 64)_ = 132.5, P < 0.0001). Column heights represent the mean while the error bars show the standard error of the mean. “***” and “****” represent P < 0.001 and P < 0.0001, respectively.

Treatment condition also had a significant effect on the moisture content (Sealed and infestation condition: F_(3, 64)_ = 147.3, P < 0.0001; Flour type: F_(1, 64)_ = 132.5, P < 0.0001) (Fig 4). Moisture content remained the same in the sealed bottles, even in those infested with RFB. The unsealed infested bottles showed significantly higher moisture levels in both maize flour (7.65 ± 0.22% (v/v)) and wheat flour (14.27 ± 0.69% (v/v)). We observed water droplets on the inside walls of unsealed infested wheat flour bottles after 50 days, which was consistent with the high moisture content in that treatment.

## Discussion

Storage in airtight containers arrests flour mass loss and the buildup of moisture that otherwise occurs when insects and a supply of oxygen are present in the bottles. When oxygen could pass freely in and out of the bottles, as was the case with the unsealed ones, we observed greatly increased populations of *T*. *castaneum* larvae and adults (wheat) and larvae, though not adults (maize). The presence of active insect populations reduced flour mass. When the bottles were hermetically sealed, the infesting population of beetles never developed beyond the first generation.

The rapid decline in oxygen is a major factor contributing to the protective effect of sealed bottles. These sealed bottles function similarly to larger hermetic containers such as hermetic bags [20]. Pattern of oxygen decline is comparable to that seen with other hermetic systems where the rate of decline is dependent on the size of the population [22,23]. While *T*. *castaneum* adults survived for some days after the bottles were sealed, all were immobile or dead within two weeks. This observation is consistent with Zhang et al. [24] who observed that 12 days under hermetic storage results in 100% RFB mortality and confirms that hermetic environments are sufficient for controlling insect pests [11,20,25]. The gradual increase in oxygen levels after an initial decrease in infested sealed bottles with both types of flour is a pattern often observed in grain stored in hermetic containers such as PICS bags [26]. While there is no clear explanation for this increase in oxygen, we speculate that this might have been due to slower leaks from the cap of the bottle or that some kind of oxygen-releasing reductive metabolism was occurring within the system. Further research is needed to explain this slow increase in oxygen levels.

The change in oxygen levels in the unsealed bottles over time reflects the substantial oxygen demands of large insect populations. High rates of population growth significantly reduce oxygen levels even within bottles that have continuous but restricted access to fresh air. High oxygen demand coupled with restricted supply of oxygen probably explains the marked drop we observed in oxygen levels in unsealed/infested bottles containing wheat flour (Fig 1). Respiration of the growing larval RFB population is likely the largest contributing factor to oxygen use.

The presence of RFB also appears to affect the distribution of oxygen in the bottles. Oxygen levels recorded from different locations (upper, middle and bottom) along the bottles’ length were significantly different. Oxygen molecules must diffuse between grain particles in order to penetrate into the grain mass [25]. Because of its fine and dense quality, the flour may retard the movement of oxygen molecules below the surface [27,28]. This property, together with unspecified low level oxidative processes in the flour could contribute to the hypoxic environment within the flour mass. The greater the distance from the air-flour interface, the greater the differential in oxygen level.

The high rates of insect respiration also likely caused the higher flour moisture content observed in unsealed infested bottles. During the experiment, we saw evidence of high moisture in the form of water droplets accumulating on the inside of the unsealed infested wheat flour bottles as well as areas of discolored flour where microbes carried by the beetles were presumably growing [29]. In the bottles in which the adult population did not grow in size, namely in the unsealed maize flour bottles, oxygen levels and flour moisture remained consistent with initial conditions.

The observed lack of growth of *T*. *castaneum* populations in the unsealed maize bottles was unexpected. While RFB is a known secondary pest of maize [30], its failure to grow to adulthood on maize flour diet when oxygen was available raises the question as to why. One possible explanation is differences in particle size between maize and wheat flour, with wheat having a more favorable particle size. When sifting out insects and contaminants of both flour types, we observed that the sieves caught more of the maize flour than the wheat flour. Other researchers have observed an inverse relationship between the particle-size of a food source and an insect’s ability to consume it [31]. Perhaps this quality, plus a lack of needed nutrients as indicated by the red color of larvae that fed on the maize flour, resulted in the observed failure of adult population growth in maize. Given that we did observe substantial numbers of larvae present in the maize flour, it may be that the maize flour used was less suitable as a diet or that the RFB population, whose source had been reared for generations on wheat flour, had not fully adapted to use maize flour as a food source.

Collectively, our results demonstrate that storage of wheat and maize flour in hermetic containers stops the growth of RFB populations and in the case of wheat flour reduces the amount of damage. In sealed bottles, oxygen levels drop rapidly, and no population growth occurs. In conclusion, hermetic containers can be used effectively to protect wheat and possibly other types of flour against insect damage. Farmers, consumers and food processors can safely store grain flour in hermetic sealed containers.

## References

[1] Hamed M, Khattak SU (Nuclear I for F and AT (Pakistan)). Red flour beetle: development and losses in various stored food stuffs [wheat shorts, starch, poprice, wheat flour, wheat bran, gram flour, biscuits and dry milk]. Sarhad J Agric [Internet]. 1985 [cited 2017 Jun 24]; http://agris.fao.org/agris-search/search.do?recordID=PK8700745#.WU712feUXzc.mendeley

[2] Arbogast RT. Beetles: Coleoptera In: Gorham JR, editor. Ecology and Management of Food-Industry Pests. Arlington, VA: AOAC; 1991 p. 131–76.

[3] Baloch UK. Wheat: Post-harvest Operations. Food Agric Organ United Nations. 1999;10.

[4] Navarro S. The use of modified and controlled atmospheres for the disinfestation of stored products. J Pest Sci (2004) [Internet]. 2012;85(3):301–22. Available from: 10.1007/s10340-012-0424-3

[5] World Bank. Missing Food: The Case of Postharvest Grain Losses in Sub-Saharan Africa. Washington D.C.; 2011.

[6] Fiedler JL, Afidra R, Mugambi G, Tehinse J, Kabaghe G, Zulu R, et al Maize flour fortification in Africa: markets, feasibility, coverage, and costs. Ann N Y Acad Sci [Internet]. 2014 4 1 [cited 2017 Jun 24];1312(1):26–39. Available from: http://doi.wiley.com/10.1111/nyas.12266 24102661 10.1111/nyas.12266

[7] Cheng W, Lei J, Ahn J-E, Liu T-X, Zhu-Salzman K. Effects of decreased O_2_ and elevated CO_2_ on survival, development, and gene expression in cowpea bruchids. J Insect Physiol [Internet]. 2012 6 [cited 2017 Jun 24];58(6):792–800. Available from: http://linkinghub.elsevier.com/retrieve/pii/S0022191012000388 22387498 10.1016/j.jinsphys.2012.02.005

[8] UNEP. Montreal Protocol on Substances that deplete the ozone layer: Report of the Methyl bromide Technical Options Committee. Nairobi, Kenya; 2002.

[9] UNEP. Handbook for the Montreal Protocol on Substances that Deplete the Ozone Layer 7th ed Nairobi, Kenya: Secretariat of the Vienna Convention for the protection of the Ozone Layer and the Montreal Protocol on Substances that Deplete the Ozone Layer; 2006.

[10] Fields PG, White NDG. Alternatives to methyl bromide treatments for stored-product and quarantine insects. Annu Rev Entomol [Internet]. 2002 1 1;47(1):331–59. Available from: 10.1146/annurev.ento.47.091201.145217 11729078

[11] Navarro S, Timlick B, Demianyk CJ, White ND. Controlled and modified atmospheres Stored Product Protection. Manhattan, KS: Kansas State University; 2012 p. 191–201.

[12] Pimentel MAG, Faroni LRD, Guedes RNC, Sousa AH, Tótola MR. Phosphine resistance in Brazilian populations of *Sitophilus zeamais* Motschulsky (Coleoptera: Curculionidae). J Stored Prod Res [Internet]. 2009 1 [cited 2017 Jun 24];45(1):71–4. Available from: http://linkinghub.elsevier.com/retrieve/pii/S0022474X08000775

[13] Benhalima H, Chaudhry MQ, Mills KA, Price NR. Phosphine resistance in stored-product insects collected from various grain storage facilities in Morocco. J Stored Prod Res [Internet]. 2004 1 [cited 2017 Jun 24];40(3):241–9. Available from: http://linkinghub.elsevier.com/retrieve/pii/S0022474X03000122

[14] Abdolmaleki A, Safavi SA, Safaralizadeh MH, Allahvaisi S, Sadeghi GR. Lethal effects of low atmosphere pressures on various developmental stages of *Tribolium castaneum* (Herbst) and *Ephestia kuehniella* (Zeller) under laboratory conditions. Egypt J Biol Pest Control. 2011;21(2):159–63.

[15] Navarro S. Modified atmospheres for the control of stored-product insects and mites In: Heaps JW, editor. Insect Management for Food Storage and Processing. 2nd ed St. Paul, MN: AACC International; 2006 p. 105–46.

[16] Banks HJ, Annis PC. Comparative advantages of high CO_2_ and low O_2_ types of controlled atmospheres for grain storage In: Calderon M, Barkai-Golan R, editors. Food Preservation by Modified Atmospheres. Florida: CRC Press; 1990 p. 93–122.

[17] Fleurat-Lessard F. Effect of modified atmospheres on insects and mites infesting stored products In: Calderon M, Barkai-Golan R, editors. Food Preservation by Modified Atmospheres. Florida: CRC Press; 1990 p. 21–38.

[18] Sanon A, Dabire-Binso LC, Ba NM. Triple-bagging of cowpeas within high density polyethylene bags to control the cowpea beetle *Callosobruchus maculatus* F. (Coleoptera: Bruchidae). J Stored Prod Res [Internet]. 2011 7 [cited 2017 Jun 24];47(3):210–5. Available from: http://linkinghub.elsevier.com/retrieve/pii/S0022474X11000282

[19] Bailey SW. Air-tight storage of grain; its effect on insect pests-IV *Rhyzopertha dominica* (F.) and some other Coleoptera that infest stored grain. J Stored Prod Res [Internet]. 1965 9 [cited 2017 Jun 24];1(1):25–33. Available from: http://linkinghub.elsevier.com/retrieve/pii/0022474X65900056

[20] Murdock LL, Margam V, Baoua I, Balfe S, Shade RE. Death by desiccation: Effects of hermetic storage on cowpea bruchids. J Stored Prod Res [Internet]. 2012 4 [cited 2017 Jun 24];49:166–70. Available from: http://linkinghub.elsevier.com/retrieve/pii/S0022474X12000033

[21] International A. No Title. In: Approved Methods of Analysis. 11th ed. St. Paul, MN: AACC International;

[22] Yakubu A, Bern CJ, Coats JR, Bailey TB. Hermetic on-farm storage for maize weevil control in East Africa. African J Agric Res [Internet]. 2011 7 18 [cited 2017 Jun 25];6(14):3311–9. Available from: http://www.academicjournals.org/journal/AJAR/article-abstract/4AC63F739267#.WU7_tYsE8Ag.mendeley

[23] Williams SB, Murdock LL, Baributsa D. Safe storage of maize in alternative hermetic containers. J Stored Prod Res [Internet]. 2017 [cited 2017 Jun 24];71:125–9. Available from: http://www.sciencedirect.com/science/article/pii/S0022474X16301618

[24] Zhang C, Rosentrater KA, Bern CJ. Laboratory-scale hermetic storage of wheat and maize against the infestation of red flour beetle (Tribolium castaneum) and maize weevil (Sitophilus zeamais) [Internet]. 2015 ASABE Annual International Meeting. St. Joseph, Mich.: ASABE; 2015. p. 1. http://elibrary.asabe.org/abstract.asp?aid=45860&t=5

[25] Murdock LL, Baributsa D, Lowenberg-DeBoer J. Special Issue on hermetic storage. J Stored Prod Res. 2014 7;58(SI):1–2.

[26] Baoua IB, Margam V, Amadou L, Murdock LL. Performance of triple bagging hermetic technology for postharvest storage of cowpea grain in Niger. J Stored Prod Res [Internet]. 2012;51:81–5. Available from: 10.1016/j.jspr.2012.07.003

[27] Flingelli G, Klementz DW. Laboratory fumigation of wheat flour with sulfuryl fluoride—penetration and fluoride residues. Proceedings 9th Int. Conf. on Controlled Atmosphere and Fumigation in Stored Products. Navarro S, Banks HJ, Jayas DS, Bell CH, Noyes RT, Ferizli AG, et al., editors. Antalya, Turkey: ARBER Professional Congress Services; 15–19 October, 2012. 172–177 p.

[28] Cenkowski S, Dexter JE, Scanlon MG. Mechanical compaction of flour: The effect of storage temperature on dough rheological properties. Can Agric Eng. 2000;42(1):1–17.

[29] Bosly HA, Kawanna MA. Fungi species and red flour beetle in stored wheat flour under Jazan region conditions. Toxicol Ind Health [Internet]. 2012 8 17;30(4):304–10. Available from: 10.1177/0748233712457449 22903175

[30] Subramanyam B, Nelson J. Profile of a Prolific Pest: Red Flour Beetle [Internet]. Whitford M, editor. Vol. 67, Pest control. Duluth, MN: Advanstar Communications; 1999 [cited 2017 Jun 24]. http://cat.inist.fr/?aModele=afficheN&cpsidt=10042629

[31] Fardisi M, Mason LJ, Ileleji KE. Development and fecundity rate of *Tribolium castaneum* (Herbst) on Distillers Dried Grains with Solubles. J Stored Prod Res [Internet]. 2013 1 [cited 2017 Jun 24];52:74–7. Available from: http://linkinghub.elsevier.com/retrieve/pii/S0022474X12000859

